# Supplementary material for: A systematic review on the prognostic role of radiologically-proven sarcopenia on the clinical outcomes of patients with acute pancreatitis
Source: PLoS One. 2025 Apr 29;20(4):e0322409. doi: 10.1371/journal.pone.0322409 (PMC12040213; doi:10.1371/journal.pone.0322409)
Supplement: S1 Appendix — (DOCX) [file pone.0322409.s002.docx]

**Appendix**

**Appendix A: Search strategy for databases and trial registries**

Ovid MEDLINE(R) ALL <1946 to July 05, 2024>

1 Pancreatitis/

2 pancreatitis.tw,kf.

3 exp Tomography, X-Ray Computed/

4 ((CAT or CT) adj2 scan).tw,kf.

5 (computed adj3 tomograph*).tw,kf.

6 3 or 4 or 5

7 1 or 2

8 6 and 7

9 Sarcopenia/

10 sarcopen*.tw,kf.

11 (muscle adj2 (atroph* or wast*)).tw,kf.

12 ((reduc* or loss* or declin*) adj3 muscle).tw,kf.

13 9 or 10 or 11 or 12

14 8 and 13

15 7 and 13

Embase <1974 to 2024 July 05>

1 Pancreatitis/

2 pancreatitis.tw,kf.

3 exp Tomography, X-Ray Computed/

4 ((CAT or CT) adj2 scan).tw,kf.

5 (computed adj3 tomograph*).tw,kf.

6 3 or 4 or 5

7 1 or 2

8 6 and 7

9 Sarcopenia/

10 sarcopen*.tw,kf.

11 (muscle adj2 (atroph* or wast*)).tw,kf.

12 ((reduc* or loss* or declin*) adj3 muscle).tw,kf.

13 9 or 10 or 11 or 12

14 8 and 13

15 7 and 13

16 limit 15 to embase

17 limit 16 to human

Cochrane central and WHO ICTRP

#1 pancreatitis

#2 sarcopen* or (muscle* NEAR/3 (atrop* or loss* or reduc* or declin* or wast*))

#3 #1 #2 22
