# Supplementary material for: A systematic review on the prognostic role of radiologically-proven sarcopenia on the clinical outcomes of patients with acute pancreatitis
Source: PLoS One. 2025 Apr 29;20(4):e0322409. doi: 10.1371/journal.pone.0322409 (PMC12040213; doi:10.1371/journal.pone.0322409)
Supplement: S1 Table — (DOCX) [file pone.0322409.s003.docx]

**S3 Table.** Table of included and excluded studies

| **Author** | **Year** | **Article title** | **Included (Y/N)** | **Reason for exclusion (if applicable)** |
| --- | --- | --- | --- | --- |
| Moreira et al. | 2022 | Visceral Adipose Tissue Influence on Health Problem Development and Its Relationship with Serum Biochemical Parameters in Middle-Aged and Older Adults: A Literature Review | N | Incorrect study design |
| Hasse et al. | 2024 | Nutrition challenges following total pancreatectomy with islet autotransplantation | N | Incorrect patient population |
| Zhao et al. | 2015 | Changes of amount and ultrastructure of interstitial cells of Cajal in rats with severe acute pancreatitis and their importance. | N | Non-human study |
| Leverve et al. | 1984 | Effect of parenteral nutrition on muscle amino acid output and 3-methylhistidine excretion in septic patients. | N | Incorrect patient population |
| Cederholm et al. | 2020 | Diagnosis of malnutrition in patients with gastrointestinal diseases: recent observations from a Global Leadership Initiative on Malnutrition perspective. | N | Incorrect patient population |
| Bischoff et al. | 2022 | European guideline on obesity care in patients with gastrointestinal and liver diseases - Joint European Society for Clinical Nutrition and Metabolism / United European Gastroenterology guideline. | N | Incorrect study design |
| Sugiura et al. | 2024 | Preoperative risk factors for skeletal muscle mass loss in patients with biliary tract cancer. | N | Incorrect patient population |
| Tsuji et al. | 1991 | Alcoholic dementia and divalent ions: derangement of calcium-homeostasis | N | Incorrect patient population |
| Chan et al. | 2021 | Elderly patients (>= 80 years) with acute calculous cholangitis have similar outcomes as non-elderly patients (< 80 years): Propensity score-matched analysis. | N | Incorrect patient population |
| Ozola-Zalite et al. | 2019 | A Clinical Feasible Method for Computed Tomography-Based Assessment of Sarcopenia in Patients With Chronic Pancreatitis. | N | Incorrect patient population |
| Bundred et al. | 2022 | Systematic review of sarcopenia in chronic pancreatitis: prevalence, impact on surgical outcomes, and survival. | N | Incorrect study design |
| Hebuterne et al. | 2008 | Artificial nutrition in acute pancreatitis | N | Incorrect comparator |
| Fujii et al. | 2022 | Cancer cachexia as a determinant of efficacy of first-line pembrolizumab in patients with advanced non-small cell lung cancer | N | Incorrect patient population |
| Gondos et al. | 1977 | Duodenal compression defect and the "superior mesenteric artery syndrome" | N | Incorrect patient population |
| Maev et al. | 2020 | The prevalence of sarcopenia in patients with chronic pancreatitis: a meta-analysis | N | Incorrect study design |
| Kozlova et al. | 2021 | Osteosarcopenia in chronic pancreatitis | N | Incorrect study design |
| Li et al. | 2023 | Computed tomography-based body composition is associated with adverse clinical outcomes among older patients with sepsis in the emergency department | N | Incorrect patient population |
| Liu et al. | 2023 | Safety and Tolerance of Umbilical Cord Mesenchymal Stem Cells in Patients With Acute Respiratory Distress Syndrome | N | Incorrect patient population |
| Molero et al. | 2024 | Chronic pancreatitis for the clinician: complications and special forms of the disease. Interdisciplinary position paper of the Catalan Society of Digestology (SCD) and the Catalan Pancreatic Society (SCPanc) | N | Incorrect study design |
| Bischoff et al. | 2023 | Practical guideline on obesity care in patients with gastrointestinal and liver diseases - Joint ESPEN/UEG guideline. | N | Incorrect study design |
| Sun et al. | 2024 | The clinical significance of sarcopenia in patients with hepatocellular carcinoma treated with lenvatinib and PD-1 inhibitors | N | Incorrect patient population |
| Dominguez-Munoz et al. | 2019 | Management of pancreatic exocrine insufficiency | N | Incorrect study design |
| Hirose et al. | 2022 | Measurement of excitation-contraction coupling time in critical illness myopathy | N | Incorrect study design |
| Monaghan et al. | 2022 | Physical activity for chronic pancreatitis: a systematic review. | N | Incorrect study design |
| Fasullo et al. | 2022 | Sarcopenia in Chronic Pancreatitis - Prevalence, Diagnosis, Mechanisms and Potential Therapies. | N | Incorrect patient population |
| Olsen et al. | 2019 | Sarcopenia associates with increased hospitalization rates and reduced survival in patients with chronic pancreatitis. | N | Incorrect patient population |
| Jalal et al. | 2022 | Identification of "Digital Sarcopenia" Can Aid the Detection of Pancreatic Exocrine Insufficiency and Malnutrition Assessment in Patients with Suspected Pancreatic Pathology. | N | Incorrect outcomes |
| Wyles et al. | 2014 | All-oral combination of ledipasvir, vedroprevir, tegobuvir, and ribavirin in treatment-naïve patients with genotype 1 HCV infection | N | Incorrect patient population |
| Duggan et al. | 2017 | Negotiating the complexities of exocrine and endocrine dysfunction in chronic pancreatitis. | N | Incorrect patient population |
| Joker-Jensen et al. | 2020 | Micronutrient deficits in patients with chronic pancreatitis: prevalence, risk factors and pitfalls. | N | Incorrect comparator |
| Kim et al. | 2015 | FGF21 as a mediator of adaptive responses to stress and metabolic benefits of anti-diabetic drugs. | N | Incorrect comparator |
| Sun et al. | 2024 | Nutrition therapy in critically ill patients with severe acute pancreatitis | N | Incorrect comparator |
| Dominguez-Munoz et al. | 2018 | Nutritional Therapy in Chronic Pancreatitis. | N | Incorrect patient population |
| Haque et al. | 2002 | Post-mortem findings in familial partial lipodystrophy, Dunnigan variety. | N | Incorrect patient population |
| Sobral-Oliveira et al. | 2011 | Nutritional profile of asymptomatic alcoholic patients | N | Incorrect comparator |
| Thompson et al. | 2016 | Treatment with ETC-1002 alone and in combination with ezetimibe lowers LDL cholesterol in hypercholesterolemic patients with or without statin intolerance | N | Incorrect intervention |
| Kuan et al. | 2016 | Prevalence and Impact of Sarcopenia in Chronic Pancreatitis: A Review of the Literature. | N | Incorrect patient population |
| Emori et al. | 2024 | Impact of sarcopenia in biliopancreatic diseases and endoscopy | N | Incorrect study design |
| Anonymous | 2015 | Abstracts of the 37th ESPEN Congress | N | Incorrect study design |
| Saito et al. | 2023 | WONDER-02: plastic stent vs. lumen-apposing metal stent for endoscopic ultrasound-guided drainage of pancreatic pseudocysts: a multicentre randomised non-inferiority trial | N | Incorrect study design |
| Maev et al. | 2021 | Exocrine pancreas insufficiency: clinical significance and approaches to correction from evidence medicine | N | Incorrect patient population |
| Furbetta et al. | 2021 | Perioperative Nutritional Aspects in Total Pancreatectomy: A Comprehensive Review of the Literature. | N | Incorrect study design |
| Paehiala et al. | 2023 | Osteoporosis and sarcopenia are common and insufficiently diagnosed among chronic pancreatitis patients. | N | Incorrect patient population |
| Naver et al. | 2020 | Energy expenditure and loss of muscle and fat mass in patients with walled-off pancreatic necrosis: A prospective study. | N | Incorrect patient population |
| Tapper et al. | 2023 | Diagnosis and Management of Cirrhosis and Its Complications: A Review | N | Incorrect study design |
| Stollberger et al. | 2023 | Adverse events and drug-drug interactions of sodium glucose co-transporter 2 inhibitors in patients treated for heart failure. | N | Incorrect patient population |
| Pittock et al. | 2023 | A double-blind randomised placebo-controlled evaluation of three doses of botulinum toxin type A (Dysport) in the treatment of spastic equinovarus deformity after stroke | N | Incorrect patient population |
| Akula et al. | 2021 | A Prospective Review of Preoperative Nutritional Status and Its Influence on the Outcome of Abdominal Surgery. | N | Incorrect study design |
| Alipour et al. | 2021 | The assessment of sarcopenia using psoas muscle thickness per height is not predictive of post-operative complications in IBD. | N | Incorrect patient population |
| Rasch et al. | 2016 | Chronic pancreatitis: Do serum biomarkers provide an association with an inflammageing phenotype? | N | Incorrect patient population |
| Zhou et al. | 2023 | Effectiveness of neuromuscular electrical stimulation in severe acute pancreatitis complicated patients with acute respiratory distress syndrome: study protocol for a randomized controlled trial. | N | Incorrect comparator |
| Petrov et al. | 2023 | Metabolic Sequelae: The Pancreatitis Zeitgeist of the 21st Century. | N | Incorrect comparator |
| Anonymous | 2013 | Abstracts of the 35th ESPEN Congress | N | Incorrect study design |
| O’Brien et al. | 2019 | Chronic Pancreatitis and Nutrition Therapy | N | Incorrect patient population |
| Sato et al. | 2022 | Immediate necrosectomy vs. step-up approach after EUS-guided drainage of walled-off necrosis: a multicenter randomized controlled trial | N | Incorrect intervention |
| Trikudanathan et al. | 2020 | Pre-operative Sarcopenia Predicts Low Islet Cell Yield Following Total Pancreatectomy with Islet Autotransplantation for Chronic Pancreatitis. | N | Incorrect patient population |
| Ishii et al. | 2021 | Impact of the preoperative body composition indexes on intraoperative blood loss in patients undergoing pancreatoduodenectomy | N | Incorrect patient population |
| Cha et al. | 2021 | Development of a nutritional index to evaluate the effectiveness of total parenteral nutrition during the early postoperative period after pancreaticoduodenectomy. | N | Incorrect patient population |
| Kataoka et al. | 2024 | mpact of sarcopenia on biliary drainage during neoadjuvant therapy for pancreatic cancer | N | Incorrect patient population |
| Donlon et al. | 2024 | FLOT-regimen Chemotherapy and Transthoracic en bloc Resection for Esophageal and Junctional Adenocarcinoma | N | Incorrect patient population |
| Hecker et al. | 2016 | Acute abdominal compartment syndrome: current diagnostic and therapeutic options | N | Incorrect comparator |
| Gossmann et al. | 1975 | Osteopathies and calcium absorption in chronic liver diseases | N | Incorrect patient population |
| Capurso et al. | 2023 | Managing complications of chronic pancreatitis: a guide for the gastroenterologist. | N | Incorrect patient population |
| Allen et al. | 2019 | Enteral Nutrition in the Mechanically Ventilated Patient | N | Incorrect comparator |
| Shintakuya et al. | 2017 | Sarcopenia is closely associated with pancreatic exocrine insufficiency in patients with pancreatic disease. | N | Incorrect outcomes |
| Yoh et al. | 2018 | Clinical influence of exercise therapy on sarcopenia in patients with chronic pancreatitis: a study protocol for a randomised controlled trial. | N | Incorrect patient population |
| Zhou et al. | 2011 | Damage of the interstitial cells of Cajal and myenteric neurons causing ileus in acute necrotizing pancreatitis rats. | N | Non-human study |
| Box et al. | 2021 | Preoperative anthropomorphic radiographic measurements can predict postoperative pancreatic fistula formation following pancreatoduodenectomy. | N | Incorrect patient population |
| Edwards et al. | 2024 | A case report of pancreatic exocrine insufficiency in a patient with Parkinson's disease: A coincidence or is there more to it than meets the eye? | N | Incorrect study design |
| Lemann et al. | 2006 | Infliximab plus azathioprine for steroid-dependent Crohn's disease patients: a randomized placebo-controlled trial | N | Incorrect patient population |
| Dominguez-Munoz et al. | 2018 | Diagnosis and treatment of pancreatic exocrine insufficiency. | N | Incorrect comparator |
| Sato et al. | 2016 | Impact of preoperative hand grip strength on morbidity following gastric cancer surgery | N | Incorrect patient population |
| Kim et al. | 2008 | Risk factors of postoperative anastomotic stricture after excision of choledochal cysts with hepaticojejunostomy. | N | Incorrect patient population |
| Wiese et al. | 2022 | Malnutrition Is Highly Prevalent in Patients With Chronic Pancreatitis and Characterized by Loss of Skeletal Muscle Mass but Absence of Impaired Physical Function. | N | Incorrect patient population |
| Nauheim et al. | 2022 | Preoperative sarcopenia is a negative predictor for enhanced postoperative recovery after pancreaticoduodenectomy. | N | Incorrect patient population |
| Petrov et al. | 2019 | Skeletal muscle: A new piece in the pancreatitis puzzle | N | Incorrect study design |
| Martin et al. | 2024 | Explanatory sequential mixed-methods approach to understand how registered dietitians implemented computed tomography skeletal muscle assessments in clinical practice | N | Incorrect intervention |
| Alshadwi et al. | 2013 | Nutritional considerations for head and neck cancer patients: A review of the literature | N | Incorrect patient population |
| Oyebola et al. | 2023 | Sarcopenia: An Assessment into the Prevalence and Disease Burden in Chronic Pancreatitis Patients. | N | Incorrect patient population |
| Mello et al. | 2017 | Electrical stimulation in cirrhotic in peripheral muscle strength and exercise capacity. | N | Incorrect patient population |
| Bischoff et al. | 2022 | European guideline on obesity care in patients with gastrointestinal and liver diseases - Joint ESPEN/UEG guideline. | N | Incorrect study design |
| Nikolic et al. | 2019 | Chronic pancreatitis and the heart disease: Still terra incognita? | N | Incorrect patient population |
| Miyoshi et al. | 1993 | A case of motor neuropathy with pyramidal sign due to prolonged administration of high dose of pancuronium bromide (Myoblock) | N | Incorrect intervention |
| Kuchay et al. | 2021 | Effect of Dapagliflozin vs Sitagliptin on Liver Fat Accumulation and Body Composition in Patients With Diabetes Mellitus and Liver Transplantation | N | Incorrect patient population |
| Zhang et al. | 2021 | Skeletal-muscle index predicts survival after percutaneous transhepatic biliary drainage for obstructive jaundice due to perihilar cholangiocarcinoma | N | Incorrect patient population |
| Kizilarslanoglu et al. | 2016 | Letter to the Editor on the Korean National Health and Nutrition Examination Survey | N | Incorrect study design |
| Shakhbazov et al. | 2020 | Morphometry of adipose tissue for prediction of the outcomes of total pancreatectomy with pancreatic islets autotransplantation in patients with chronic pancreatitis | N | Incorrect patient population |
| Donlon et al. | 2022 | Cross versus flot regimens in esophageal and esophagogastric junction adenocarcinoma a propensity-matched comparison | N | Incorrect patient population |
| Facciorusso et a. | 2020 | Sarcopenia represents a negative prognostic factor in pancreatic cancer patients undergoing EUS celiac plexus neurolysis | N | Incorrect patient population |
| Onesti et al. | 2016 | Sarcopenia and survival in patients undergoing pancreatic resection | N | Incorrect patient population |
| Fujii et al. | 2020 | Cancer cachexia reduces the efficacy of nivolumab treatment in patients with advanced gastric cancer | N | Incorrect patient population |
| Kumar et al. | 2017 | Visceral Adipose Tissue as a Risk Factor for Diabetes Mellitus in Patients with Chronic Pancreatitis: A Cross-sectional, Observational Study | N | Incorrect patient population |
| Brandi et al. | 2022 | An Imaging Overview of COVID-19 ARDS in ICU Patients and Its Complications: A Pictorial Review. | N | Incorrect patient population |
| Marotto et al. | 2020 | Extra-intestinal manifestations of inflammatory bowel diseases | N | Incorrect patient population |
| Simonsen et al. | 2021 | Assessment of sarcopenia in patients with upper gastrointestinal tumors: Prevalence and agreement between computed tomography and dual-energy x-ray absorptiometry | N | Incorrect patient population |
| Chorley et al. | 2024 | Successful adult domino living donor liver transplantation in methylmalonic acidemia: case report | N | Incorrect patient population |
| Iwashita et al. | 2023 | Supportive treatment during the periprocedural period of endoscopic treatment for pancreatic fluid collections: a critical review of current knowledge and future perspectives. | N | Incorrect study design |
| Matsumoto et al. | 2024 | Skeletal muscle mass and function are affected by pancreatic atrophy, pancreatic exocrine insufficiency and poor nutritional status in patients with chronic pancreatitis. | N | Incorrect patient population |
| Ehlers et al. | 2020 | Preclinical insights into the gut-skeletal muscle axis in chronic gastrointestinal diseases. | N | Incorrect study design |
| Modesto et al. | 2020 | Reduced Skeletal Muscle Volume and Increased Skeletal Muscle Fat Deposition Characterize Diabetes in Individuals after Pancreatitis: A Magnetic Resonance Imaging Study. | N | Incorrect comparator |
| Bieliuniene et al. | 2019 | CT- and MRI-Based Assessment of Body Composition and Pancreatic Fibrosis Reveals High Incidence of Clinically Significant Metabolic Changes That Affect the Quality of Life and Treatment Outcomes of Patients with Chronic Pancreatitis and Pancreatic Cancer. | N | Incorrect patient population |
| Yoh et al. | 2017 | Effect of exercise on sarcopenia in patients with chronic pancreatitis | N | Incorrect patient population |
| Modesto et al. | 2020 | Psoas muscle size as a magnetic resonance imaging biomarker of progression of pancreatitis | N | Incorrect comparator |
| Yoon et al. | 2017 | Impact of body fat and muscle distribution on severity of acute pancreatitis. | N | Incorrect comparator – sarcopenia not defined |
| Akturk et al. | 2021 | The Effects of the Fat Distribution of Body, Skeletal Muscle Mass and Muscle Quality on Acute Pancreatitis Severity: A Retrospective Cross-Sectional Study. | N | Incorrect comparator – outcomes not assessed |
| Kolck et al. | 2024 | Opportunistic screening for long-term muscle wasting in critically ill patients: insights from an acute pancreatitis cohort. | N | Incorrect comparator – outcomes not assessed |
| Van Grinsven et al. | 2017 | The Association of Computed Tomography-Assessed Body Composition with Mortality in Patients with Necrotizing Pancreatitis. | N | Incorrect comparator – psoas muscle index classified into tertiles |
| Chaingneau et al. | 2023 | Impact of sarcopenic obesity on predicting the severity of acute pancreatitis. | N | Incorrect comparator – outcomes not assessed |
| Farquhar et al. | 2023 | Sarcopenia and Sarcopenic Obesity on Body Composition Analysis is a Significant Predictor of Mortality in Severe Acute Pancreatitis: A Longitudinal Observational Study. | Y | N/A - Included |
| Yee et al. | 2021 | Dynamic frailty: Objective physiological assessment to guide management in necrotizing pancreatitis. | Y | N/A - Included |
| Yu et al. | 2021 | Assessment of Computed Tomography-Defined Muscle and Adipose Tissue Features in Relation to Length of Hospital Stay and Recurrence of Hypertriglyceridemic Pancreatitis. | Y | N/A- Included |
| Fu et al. | 2023 | Cutoff Value of Psoas Muscle Area as Reduced Muscle Mass and Its Association with Acute Pancreatitis in China. | Y | N/A - Included |
