# Supplementary material for: A systematic review on the prognostic role of radiologically-proven sarcopenia on the clinical outcomes of patients with acute pancreatitis
Source: PLoS One. 2025 Apr 29;20(4):e0322409. doi: 10.1371/journal.pone.0322409 (PMC12040213; doi:10.1371/journal.pone.0322409)
Supplement: S5 Table — (DOCX) [file pone.0322409.s007.docx]

**S7 Table.** Data for venous thromboembolic phenomenon of included studies

| **Author** | **Year** | **Sarcopenia**  **(events)** | **Sarcopenia**  **(total)** | **Non-sarcopenia**  **(events)** | **Non-sarcopenia**  **(total)** | **Data extracted by** |
| --- | --- | --- | --- | --- | --- | --- |
| Farquhar et al. | 2023 | 10 | 44 | 10 | 30 | Khang Duy Ricky Le, Harsh Patel |
| Fu et al. | 2023 | 3 | 29 | 4 | 241 | Khang Duy Ricky Le, Harsh Patel |
